# Evaluation of Two Resin Composites Having Different Matrix Compositions

**DOI:** 10.3390/dj8030076

**Published:** 2020-07-17

**Authors:** Tarek M. Elshazly, Christoph Bourauel, Dalia I. Sherief, Dalia I. El-Korashy

**Affiliations:** 1 Oral Technology, School of Dentistry, University of Bonn, 53111 Bonn, Germany; bourauel@uni-bonn.de; 2 Biomaterials Department, Faculty of Dentistry, Ain Shams University, 11566 Cairo, Egypt; dsherief77@gmail.com (D.I.S.); daliakorashy71@gmail.com (D.I.E.-K.)

**Keywords:** smart polymers, polymerization shrinkage, bulk-fill resin composite, microleakage

## Abstract

This study compared two resin composites with similar filler systems and different matrix compositions. The depth of cure (DoC), polymerization shrinkage, and marginal leakage were evaluated. A Filtek Bulk Fill resin composite (FB) and a Filtek Supreme resin composite (FS) were used. For the DoC and polymerization shrinkage, cylindrical specimens with different thicknesses were prepared. The DoC was attributed to the bottom/top ratios of Vickers microhardness numbers. For polymerization shrinkage, each specimen was firstly scanned using micro-computed tomography (µCT) then cured for 20 s, then for 10 s, and then for 10 s, and they were rescanned between each curing time. Data were processed using the Mimics software. For marginal leakage, standardized 5 mm cavities were prepared in 90 molars. After etching and bonding, materials were packed according to groups: FB-bulk, FB-incremental, and FS-incremental, which were cured for 20, 30, and 40 s, respectively. After thermo-cycling, teeth were stored in 1% methylene blue dye for 24 h and then sectioned and observed for dye penetration. The results showed insignificant differences in the shrinkage and leakage between the different packing techniques and curing times of both materials. In conclusion, the introduction of a novel matrix into resin composite composition enabled bulk-filling in one layer up to 5 mm deep while keeping a tolerable polymerization shrinkage.

## 1. Introduction

Polymerization reactions of most resin composites involve the rupture of the covalent aliphatic double bonds C=C in the reacting monomers and formation of single covalent bonds C–C. This is mostly accompanied by the shrinkage and reduction of the intermolecular distances by 0.3–0.4 nm between polymer chains [1]. The shrinkage induces stresses and strains in the resin composite and cavity walls, as well as the interface between them [2]. If the adhesion quality is not adequate, adhesive failure and microleakage may occur. Consequently, problems like recurrent caries, discoloration, sensitivity, and pulpal irritation may occur [3,4].

Numerous efforts have been exerted to introduce a material that could be clinically applied with a fast time-saving simple technique instead of the 2 mm incremental layering technique. Modifications in monomers’ chemistry, initiation system, and/or fillers have led to the introduction of bulk-fill resin composites [2,5,6,7,8]. The technology of bulk-fill resin composites is based on a huge dental industry that has experienced new types of monomers, such as expanding monomers [9] and ring-opening monomers [10], as well as innovative mechanisms of polymerization reactions [11]. Furthermore, the incorporation of high molecular weight monomers has become popular [12]. Additionally, the incorporation of stress-reducing modulators into the resin backbone of a resin composite could result in the slower development of the modulus of elasticity. Hence, these modulators allow for more time for stress reduction without decreasing the rate of conversion [11,13,14].

An ideal bulk-fill resin composite would be one that could be packed into a deep cavity with a high configuration factor design while still attaining a high degree of conversion throughout the whole restoration and exhibiting very little polymerization shrinkage stresses [15]. In order to evaluate the depth of cure (DoC), Asmussen [16] reported a direct correlation between material’s hardness and the curing of the resin composite. The ISO 4049 method has not been found to accurately estimate the DoC for bulk fill materials when it has been compared to the values of the DoC determined by hardness testers [17]. According to a well-accepted definition, the DoC is the adequate thickness of a layer of resin composites, where the hardness at the bottom of it is equal to at least 80% of the maximum hardness at the top of it [12].

Polymerization shrinkage could be indirectly estimated in terms of volumetric/linear shrinkage and cuspal deflections, or it could be indirectly estimated by marginal leakage evaluation. Several methods have been used to determine polymerization shrinkage, and micro-computed tomography (µCT) is one of them [18,19]. Three-dimensional µCT is a cone-beam tomography that produces high-resolution images of up to a few micrometers. It is a non-destructive method that is extensively used in the investigation of bone-density [20] and in the assessment of mineral content in dental studies [21]. Furthermore, µCT data could be used for polymerization shrinkage evaluation, either by the assessment of volumetric changes and micro-gap analysis or by the calculations of the shrinkage vector through tracing particles before and after polymerization [18,22,23].

Marginal microleakage measurements are valuable as pre-clinical screening tests [24]. The use of organic dyes as tracers is one of the oldest and most common in vitro techniques. A number of dyes, different in particle size and affinity to substrates, are used. The type of dye used is known to significantly influence microleakage results [25]. One of the most common dyes is methylene blue dye [26].

3M OralCare has introduced a bulk-fill resin composite (Filtek Bulk Fill) that has the same filler system but is different from conventional resin composites (Filtek supreme XTE) in that it has a novel matrix, as claimed by the manufacturer. This study was designed to compare both resin composites and to evaluate how far the introduction of a novel matrix composition only, without the modification of the filler system, allowed for bulk-filling of a resin composite in one layer in a cavity up to 5 mm deep while keeping a tolerable polymerization shrinkage. The comparison was done via the assessment of the depth of cure, polymerization shrinkage, and marginal leakage with different packing techniques and curing times. The depth of cure was attributed to the bottom/top ratios of Vickers microhardness numbers, polymerization shrinkage was measured using µCT scanning combined with mimics software, and marginal leakage was semi-quantitatively assessed by a methylene dye penetration test.

## 2. Materials and Methods

### 2.1. Materials

The Filtek Bulk Fill (FB) bulk-fill resin composite (shade: A2; 3M OralCare, St. Paul, MN, USA) and the Filtek Supreme XTE (FS) conventional resin composite (shade: A2 Body; 3M OralCare, St. Paul, MN, USA) were utilized. These two resin composites have nearly the same filler system, but they have different polymeric matrixes (Table 1).

### 2.2. Methods

#### 2.2.1. Depth of Cure (DoC)

A total of 90 specimens were prepared, with *n* = 10 for each experimental condition. Specimens were grouped based on the material packing technique (type of resin composite and thickness of increments) and the time of curing. In the Filtek Supreme-incremental (FS-I) group, a Filtek Supreme resin composite was applied in an incremental pattern to achieve a final thickness of 5 mm (2 + 2 + 1 mm). In the Filtek Bulk-incremental (FB-I) group, a Filtek Bulk fill resin composite was applied in an incremental pattern to achieve a final thickness of 5 mm (2 + 2 + 1 mm). Finally, in the Filtek Bulk-bulk (FB-B) group, a Filtek Bulk fill resin composite was applied in a bulk pattern with a thickness 5 mm. Each group was subdivided into 3 sub-groups according to the curing time per increment—20, 30, or 40 s.

The material was packed in a Teflon split-mold (4 mm diameter and 5 mm depth). The mold was placed on a glass slide, and a celluloid matrix was placed in between. After the packing of the resin composite, another celluloid strip was placed on the top surface and gently pressed using another glass slide to extrude the excess material. The specimens were light cured using an LED curing device (Elipar DeepCure-S, 3M OralCare, St. Paul, MN, USA), which produces blue light with a wavelength of 430–480 nm and an output intensity of 1470 mW/cm^2^, and its 10 mm diameter light guide tip was directly placed on top of the celluloid strip covering the specimen’s surface. The light intensity was checked every 3 samples with a built-in radiometer to ensure consistent light output throughout the study. The resin composite material was packed and cured according to the previously mentioned specimens’ grouping. The excess material was removed, and the specimens were polished from top and bottom with 1200-grit silicon carbide grinding paper (CarbiMet 2 Abrasive Discs, Buehler, Lake Bluff, IL, USA). The dimensions of the specimens were checked by a digital caliper (CONNEX, Dettingen, Germany).

The surface microhardness of the top and bottom surfaces of the specimens was measured with a Vickers microhardness tester (model 422A/423A, Innovatest, Maastricht, Niederland) equipped with an analogue eyepiece (40X magnification) with micrometer reading to determine the indent diagonals. The applied load was 1000 g force with a dwell time of 10 s. The average of three measurements was taken for every surface. The DoC was attributed to the bottom/top hardness number ratios.

#### 2.2.2. Polymerization Shrinkage

**Figure 1:**
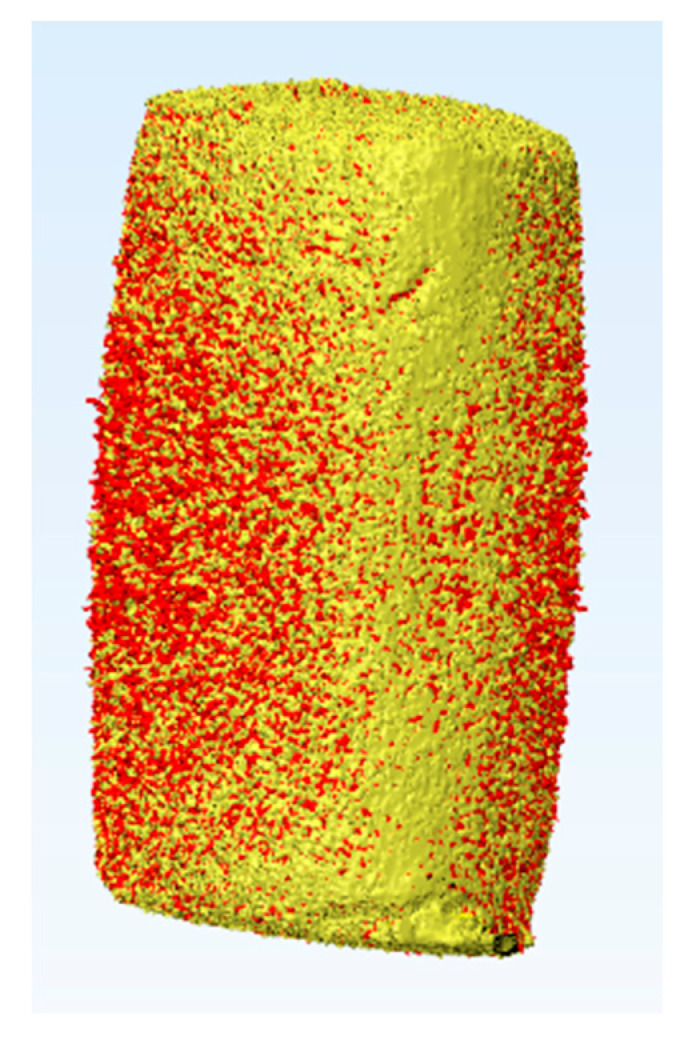
Superimposition of 3D objects created by growing a threshold region on the entire stack of scans of Filtek Bulk Fill resin composite specimen (5 mm) before curing (yellow) and after 20 s curing (red). The 3D properties of the object provided information about the volume of the specimen in µm^3^. By comparing the volume before and after curing, the percentage of the polymerization shrinkage could be calculated.

A total of 30 cylindrical specimens were prepared, 10 specimens per each packing technique (*n* = 10). The material was packed in Teflon split molds (4 mm diameter) with different thicknesses: FB-B = 5 mm, FB-I = 2 mm, and FS-I = 2 mm. After packing the material and before curing, each specimen was scanned using µCT (Skyscan 1174, Skyscan, Kontich, Belgium) at a resolution of 7.7 μm, a beam current of 800 μA, an accelerating voltage of 50 kVp, and an exposure time of 22 s, with no filters and with a rotation of 360° at a 0.5° rotation step. The average of the total number of slices was 1022, with an average scanning time nearly equal to one hour. No curing of the material during scanning was secured since the inner compartment of the equipment was totally dark. After scanning, the specimen was cured for 20 s, as done with the DoC specimens. The specimen was rescanned after curing. Then, the specimen was cured for an extra 10 s to achieve a total curing time of 30 s, and then it was rescanned. Lastly, the specimen was cured for an extra 10 s to achieve a total curing time of 40 s, and it was rescanned using the same parameters each time. The 2D data were exported as DICOM files into a 3D interactive medical image processing system (Mimics 9.0, Materialise, Leuven, Belgium). Mimics offers extended visualization and segmentation functions based on image radio-opacity thresholding. A 3D object of each specimen could be automatically created by growing a threshold region on the entire stack of scans. In addition, the superimposition of the specimen’s 3D object before and after curing could be achieved using the 3-matic tools (Figure 1). Optimal radio-opacity threshold determination was a highly-sensitive step during the determination and analysis of the 3D objects; therefore, a sensitivity test was performed to determine the best parameters for defining the correct volume of the specimens. The 3D properties of the object provided information about the volume of the specimen in µm^3^. The volume of the specimen was checked experimentally. Each specimen was a reference to itself, and the polymerization shrinkage calculation was based on the original pre-curing volume of each specimen individually. By comparing the volume before and after curing, the percentage of the polymerization shrinkage could be calculated by applying the following equation:

$$PS\;\%=\left(\left(V-V^{{}^{\circ}}\right)/V^{{}^{\circ}}\right)\times 100$$

where *PS* is polymerization shrinkage, *V* is the volume after curing, and *V*° is the volume before curing.

#### 2.2.3. Marginal Leakage

**Figure 2:**
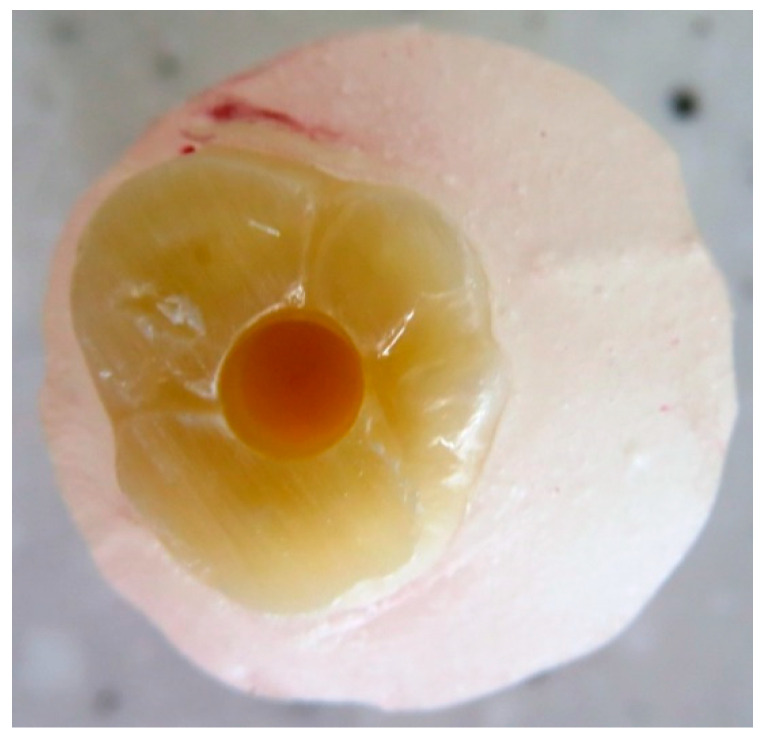
Standardized occlusal cavity with a 5 mm depth in an upper sound molar embedded in a cold-curing resin block up to directly below the cemento–enamel junction after the sealing of the roots by 2 layers of water-proof nail varnish.

A total of 90 recently extracted sound human wisdom molars, which were extracted for orthodontic or surgical purposes, were collected from the dental school of the university hospital Bonn. They were cleaned from soft tissue debris by using a manual scaler and then stored until usage in 0.5% chloramine solution at 4 °C until testing for a maximum of two months. The root apices were sealed with a resin composite, and the roots were double coated with a water-proof nail varnish; then, they were embedded in a cold-curing resin blocks (Technovit 4004, Kulzer, Hanau, Germany) up to directly below the cemento–enamel junction for the easier handling of the teeth. The occlusal surfaces of all the samples were flattened by the aid of a water-cooled diamond disc (Komet, Brasseler, Lemgo, Germany). Each tooth block was mounted on the surveying table of a drilling/milling machine (F1 model milling-machine, DeguDent/Degussa, Frankfurt, Germany) that was equipped with Kavo K9 handpiece (Kavo Dental, Biberach, Germany). The handpiece of the drilling machine was only used for a vertical movement depth of up to 5 mm and a rotation speed of 25,000 rpm in presence of copious water spray. A new drill was used for every 5 cavity preparations. One standardized cylindrical occlusal cavity, 5 mm deep, was prepared in each tooth (Figure 2) with a cylindrical 3 mm diameter carbide drill (Carbide tipped jobber drill, Garant, Hoffmann, Munich, Germany). The dimensions of the cavity were determined by the size of the drill, and the machine settings and were verified using a periodontal probe. Teeth with pulp exposure upon cavitation were discarded.

The teeth were blindly divided into 9 experimental groups: 3 main groups according to packing technique: FB-B, in which the resin composite was packed in single layer of 5 mm, and FB-I and FS-I, in which the resin composite was incrementally packed in the 5 mm cavity (2 + 2 + 1 mm) and each layer was cured individually. Each main group was subdivided into three different curing time groups (20, 30, and 40 s). Ten teeth were used for each experimental condition (*n* = 10). The enamel margins were etched with a 36% phosphoric acid gel (Scotchbond Universal Etchant Etching Gel, 3M OralCare, St. Paul, MN, USA) for 30 s (manufacturer’s instructions). The cavity was then washed with copious air/water spray for 30 s and then it was gently dried with an oil-free air. A bonding agent (Scotchbond Universal adhesive, 3M OralCare, St. Paul, MN, USA) was applied to the cavity with gentle agitation followed by 5 s of the gentle application of an oil-free air and then 10 s of light-curing (following the manufacturer’s instructions). Light-curing was achieved by using the same LED curing device of the µCT test.

**Figure 3:**
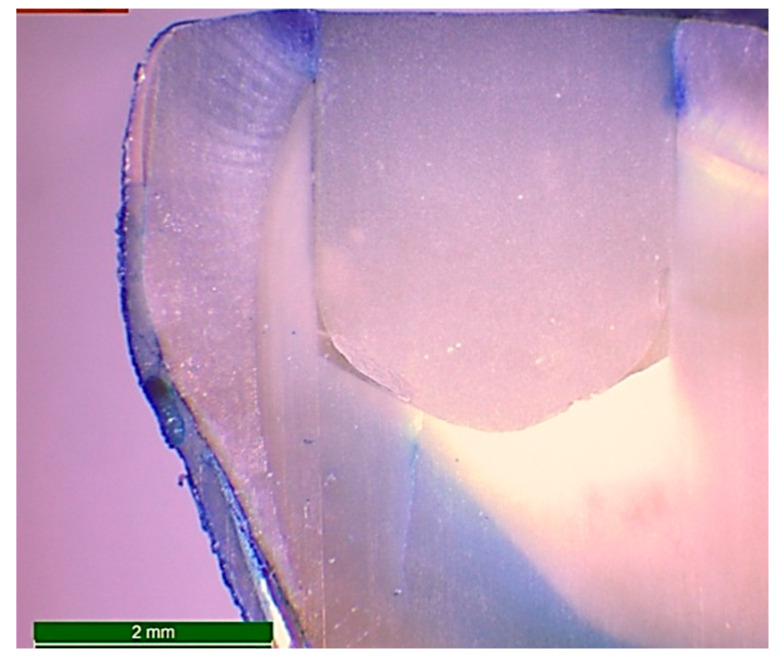
Microphotograph at 15X magnification of a longitudinal section of a restored standardized cavity in a sound molar after 5000 cycles of thermo-cycling and immersion in freshly prepared 1.0% methylene blue dye for 24 h in order to examine the marginal leakage by evaluation of the grade of dye penetration.

**Figure 4:**
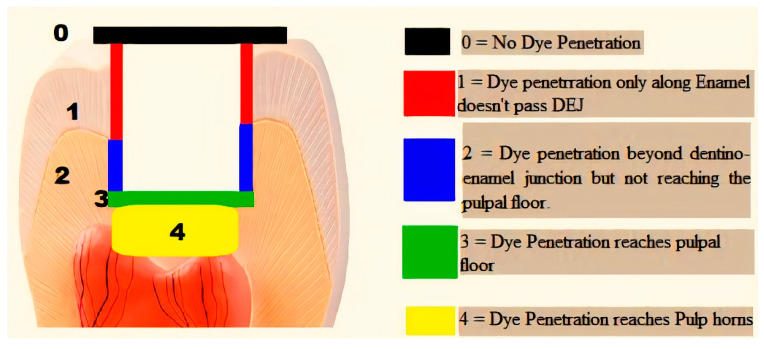
Schematic diagram representing the used scoring system of marginal leakage.

After the packing and curing of the resin composite according to the previously mentioned specimens’ grouping, the surfaces of the restorations were finished and polished using ascending grits (Soflex, 3M OralCare, St. Paul, MN, USA). Prepared specimens for microleakage testing were subjected to cycles of thermal fluctuations for the simulation of artificial aging using a thermocycler (Thermocycler THE-1100, SD Mechatronik, Germany) from 55 (±1) to 5 (±1) °C, with a dwell time of 30 s and a transfer time between each water bath of 10 s—for 5000 cycles. After the completion of thermo-cycling, the teeth were dried and double coated with a waterproof nail varnish along the whole surfaces except for 1 mm around the margins of the restoration. Then, the teeth were immersed in freshly prepared 1.0% methylene blue dye (Certistain, Merck, Darmstadt, Germany) for 24 h. The teeth were then rinsed thoroughly with running water and sectioned buccolingually along their long axes into two sections using an automated water-cooled diamond saw (EXAKT 300 CP Band System, Norderstedt, Germany). The specimens were examined for marginal leakage and dye penetration by using stereomicroscope (Wild Heerbrugg transmitted-light stand EB, Leica microsystem, Wetzlar and Mannheim, Germany) at a magnification of 15X (Figure 3). The scoring of dye penetration was done for marginal leakage (Figure 4) as follows:

❖ 0 = No dye penetration.
❖ 1 = Dye penetration is only along enamel and does not pass the dentino–enamel junction (DEJ).
❖ 2 = Penetration of the dye beyond the dentino–enamel junction but not reaching the pulpal floor.
❖ 3 = Penetration of the dye to the pulpal floor.
❖ 4 = Penetration of the dye to the pulp horn.

#### 2.2.4. Statistical Analysis

The power analysis was designed to have adequate power to apply a two-sided statistical test of the research hypothesis (null hypothesis) that there was no difference between different tested materials. According to Lima et al. [27], Kamalak et al. [19], and Orlowski et al. [28], effect size (f) was found to be 9.81. By adopting an alpha (α) level of 0.05 and a beta (β) level of 0.20 (power = 80%); the predicted sample size (n) was found to be a total of 4 samples per group. Sample size calculation was performed using G*Power version 3.1.9.4.

Statistical analysis was performed with IBM SPSS (IBM Company, Endicott, New York, USA) Statistics Version 25 for Windows. Regarding the depth of cure and polymerization shrinkage tests, the data were normally distributed, and a two-way ANOVA was used to analyze the effects of curing time, the packing technique, and their interactions. Marginal leakage data are presented as median and range values, and the effect of curing time was analyzed using Friedman’s test followed by multiple pairwise comparisons utilizing Wilcoxon signed-rank test with the Bonferroni correction. The effect of the packing technique was analyzed using the Kruskal–Wallis test followed by multiple pairwise comparisons utilizing the Mann–Whitney U test with the Bonferroni correction. The significance level was set at *p* ≤ 0.05 for all tests.

## 3. Results

The results of the DoC in the present study revealed that the hardness bottom/top ratio of both tested materials in all groups fulfilled a minimum value of 0.9. The polymerization shrinkage value range was between −1.4% (FB-bulk— cured for 20 s) and −1.9% (FS-incremental—cured for 40 s). Regarding marginal leakage, (FS-I) and (FB-I) packing techniques at a 40 s curing time were the only groups among the different groups, which recorded a marginal leakage score 4, while the FB-B packing technique group at 20 and 40 s curing times were the only groups that recorded a score of 0. No significant difference was found in polymerization shrinkage and marginal leakage values between the different packing techniques and curing times of both tested resin composite materials.

**Figure 5:**
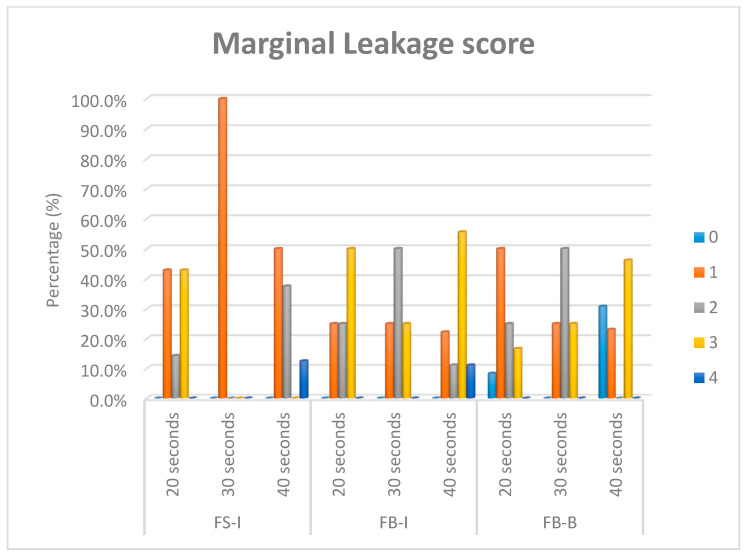
Bar chart showing the percentage (%) of marginal leakage scores for different curing times and packing techniques of Filtek Bulk-bulk (FB-B), Filtek Bulk-incremental (FB-I), and Filtek Supreme-incremental (FS-I).

Means and standard deviations (SD) for the bottom/top ratios’ Vickers hardness numbers (depth of cure) for different curing times and packing techniques are presented in Table 2. Mean and standard deviation values for polymerization shrinkage are presented in Table 3. Median and range values for marginal leakage scores for different packing techniques and curing times are presented in Table 4. The percentage (%) of marginal leakage scores for different curing times and packing techniques are presented in Figure 5.

## 4. Discussion

The DoC is a cornerstone in evaluating any newly introduced resin composite [29]. Based on ISO standards, the acceptable restoration’s DoC is its thickness when the hardness at its bottom surface is at least 80% of the hardness at its top surface [12,30,31]. However, some authors [11,29] have criticized this method because the size of the micro-indenter is considerably larger than the distance between neighboring fillers of resin composites. However, it is still a well-accepted method in the literature, especially when the hardness number is measured at more than one location on the surface and the mean is taken afterwards [12,31,32,33,34].

The Vickers microhardness tester is one of the most commonly used microhardness testers. It has the advantage of assurance of a constant value for the hardness number over a wide range of test load [35]. In the current study, a high load was used (1000 gm) because it could produce a larger impression and therefore made it easier to measure the indentation diagonal [36]. In addition, some studies of microhardness on a wide range of loads have demonstrated that the results are not constant at very low loads [37].

The results of the DoC in the present study revealed that the hardness ratio of all tested materials fulfilled a minimum value of 0.9 bottom/top ratio. This indicated an adequate polymerization throughout the whole specimen in spite of the 5 mm bulk-filling of the FB resin composite. This could be explained by the fact that FB has more pre-curing translucency and the greater penetration of curing light into the deep layer without attenuation due to the conducting adjustment of the refractive indices of the matrix/filler, as reported by the manufacturer. This is in a good agreement with studies by Alshali et al. [38] and Alrahlah et al. [34], who used a Vickers hardness tester to evaluate the DoC of different types of bulk-fill resin composites including FB. They reported that FB attained a sufficient bottom/top hardness ratio. However, Alrahlah et al. [34] reported a maximum depth of cure for FB of only 4.14 mm. This difference could be attributed to the different types of mold used in the study (stainless steel mold).

The FB-B group showed a significantly lower DoC with a 20 s curing time, as compared to the FB-I and FS-I groups, reflecting the requirement of higher amount of radiant exposure to attain an equal bottom/top ratio at a 5 mm depth [39]. A similar study by Lempel et al. [40] reported that the extended curing time of FB significantly increased the DoC, especially at the bottom surface, which consequently increased hardness. An interesting finding revealed by the current study was that the DoC of FS-I at 20 s was significantly higher than with 30 and 40 s curing times (20 > 30 = 40). Leprince et al. [29] reported that 40 s of irradiation resulted in a greater mobility of unreacted monomers, leading to more polymerization and, consequently, a higher hardness at the surface of the material, which means a lower bottom/top ratio.

A digital non-destructive full analysis of pre-cured and post-cured material can be performed in three dimensions with sufficient opacity by using µCT [18,19,22]. The µCT technique, combined with Mimics software employed in this study, resulted in the successful detection of the specimens and the accurate calculation of their volumes, which were checked experimentally. It was observed that the threshold range was little bit different from Filtek Supreme specimens and Filtek Bulk Fill specimens, which could be referred to the slight difference in filler content percentage (Table 1) [22]. Each specimen was a reference to itself, so the slight difference in volume resulting during the preparation of different specimens could be ignored.

The results of this study showed an insignificant difference in polymerization shrinkage between tested materials despite the difference in the packing techniques and curing times. That could be explained by the presence of an innovative stress-relieving mechanism. The incorporation of a novel monomer called the addition–fragmentation molecule (AFM), which cleaves during the polymerization reaction, could provide a mechanism of stress relief while maintaining the physical properties of the polymer [14]. In addition, the Filtek Bulk Fill resin composite contained modified, high molecular weight aromatic urethane dimethacrylate (AUDMA) monomers. These monomers have a lower concentration of double bonds that consequently increase the degree of conversion while maintaining a reduced polymerization shrinkage [41,42]. Despite the insignificant differences in the measured shrinkage, however, there was still an apparent and expected decreasing trend form FS-I to FB-I to FB-B.

The polymerization shrinkage values reported in this study were in agreement with many studies that have reported the polymerization shrinkage of the resin composite in the range of 1.2%–2.7% [19,43,44,45]. Junior et al. [45] reported that the polymerization shrinkage of the incrementally placed Filtek Supreme was 1.2 ± 1.0%, and the slight difference could have been caused by the difference in the layer thickness used in their study (≈1.3 mm). Kamalak et al. [19] evaluated the volumetric shrinkage of the flowable Filtek Ultimate (Supreme) and flowable Filtek Bulk Fill using µCT Skyscan 1172 and the CTAn software. They reported an insignificant difference between Filtek Supreme and Filtek Bulk Fill resin composites with values of 2.1 ± 0.8 and 2.0 ± 0.5, respectively. Kim et al. [46] measured the polymerization shrinkage using positron sensitive photo detector (PSPD) equipment, and seven different flowable resin composite resins were tested in their study. They reported that the flowable Filtek Supreme demonstrated a shrinkage value of 2.2 ± 0.7%. The difference in these reported values compared to the current study could be attributed to the use of flowable resin composites in their study [2,47]. On the other hand, the results of the current study were different from a study by Sampaio et al. [23], who reported a 5.5 ± 1.8% polymerization shrinkage of the flowable Filtek Bulk by using the µCT of different types of resin composites.

The main aim of in vitro marginal leakage tests was to predict the clinical behavior of the restoration. The use of the dye penetration test remained the most popular for in vitro microleakage evaluations, qualitatively or semi-quantitatively [24,25]. Methylene blue dye was used as a tracer in the current study. Different storage periods of the samples in the dye were reported, but 24 h was the most common period [28], and this was the choice in the present study. Nevertheless, it was stated in a review [48] that the storage period in methylene blue dye seems to have no influence on microleakage scores.

In the current study, an occlusal cavity with a cylindrical design was chosen. Several cavity designs have been used in other studies—either class V or occlusal cavities, including cylindrical, rectangular, and wedge-shaped preparations. However, the dentinal tubules in a cylindrically shaped cavity are almost aligned with the walls of the preparation compared to wedge-shaped class V preparation [49]. Moreover, in the class V cavity design, the occurrence of prismless enamel at the gingival margins is frequent. There is a limitation of resin penetration into prismless enamel, which may not provide an effective barrier to dye penetration and may affect results [50]. In addition, the use of a parallelometer ensured the attainment of standardized cavities during the study [51].

Clinically, restorations are exposed to the long-term accumulation of temperature fluctuations, which could lead to marginal deterioration. In an attempt to simulate the clinical situation, specimens were exposed to thermo-cycling from 55 (±1) to 5 (±1) °C for 5000 cycles [52]. Therefore, the application of thermal cycling during testing is expected to provide a better prediction of in vivo performance [24,53,54].

The results of marginal leakage evaluation revealed an insignificant difference in marginal leakage scores for the bulk resin composite compared to the conventional one, in spite of the difference in packing techniques. This could have been due to the approximately similar filler loadings, as well as the incorporation of stress modulator and high molecular weight monomers in the bulk-fill resin composite matrix (Table 1). The current results also revealed insignificant differences in marginal leakage between different curing times with different packing techniques. This is not in harmony with the understanding of the polymerization reaction kinetics of most resin composites in which a longer curing time leads to the greater formation of free radicals, which means a higher rate of chain growth and higher conversion of double bonds—both of which involve volumetric shrinkage [10,55,56]. However, this conflict could be explained by the presence of stress relieving mechanism in FB, as mentioned before.

These results contradict those of a study done by Kim et al. [46], who reported less microleakage for flowable FB than for flowable FS. They attributed their results to the presence of a bisphenol A polyethylene glycol diether dimethacrylate (Bis-EMA) monomer in FB that usually exhibits a higher degree of conversion and a lower amount of polymerization shrinkage than the typical bisphenol-A glycidyl dimethacrylate/triethylene glycol dimethacrylate (Bis-GMA/TEGDMA) combination contained in the flowable FS. Zorzin et al. [6] also found that the flowable FB showed less shrinkage than the flowable FS, and they attributed that to presence of a larger amount of TEGDMA in FS (5–10 wt%) than in FB (<1 wt%), which reduced the viscosity of resin composites and hence increased polymerization shrinkage.

In an agreement with Furness et al. [15], it was obvious that dye penetration into the bonded interface had random different behaviors among different materials. In addition, there was a notable gap formation at the pulpal floor of all restorations. Additionally, the observation of dye rush into the dentinal tubules in many sections may be attributed to the position of the micro-gap formation along the interface. If such a formation occurs between the restoration and the hybrid layer, a localized pooling of the dye may be expected, since the tubules remain sealed. However, if the micro-gap occurs between the hybrid layer and the underlying dentine, dye penetration would be expected.

The present study had the limitations of measuring the volumetric polymerization shrinkage of free unbonded resin composite specimens. In addition, the semi-quantitative evaluation of marginal leakage can only act as a precursor to in vivo studies. Furthermore, although methylene blue dye is a commonly used tracer, it has a small particle size and as such may lead to an overestimation of the relevance of its infiltration [26] and give higher microleakage scores than other microscopic evaluations [57]. Further studies are recommended regarding the directions of shrinkage and the correlation between the degree of conversion and the polymerization shrinkage. Clinical studies are suggested.

## 5. Conclusions

The introduction of a novel matrix into a resin composite, without the modification of the filler system, enabled the bulk-filling in one layer up to 5 mm deep while keeping a tolerable polymerization shrinkage.

## Figures and Tables

**Table 1:** Materials used in the study, their lot number, and composition.

Material	Lot No.	Composition ^1^
Filtek Bulk Fill posterior restorative Shade: A2	N701975	Fillers: non-agglomerated nanosilica of 20 nm filler size and agglomerated zirconia/silica nanocluster with a size of 5–20 nm. The filler loading was 76.5 wt% (58.5% by volume). Organic matrix: Bisphenol-A glycidyl dimethacrylate (Bis-GMA) (1–10 wt%), urethane dimethacrylate (UDMA) (10–20 wt%), triethylene glycol dimethacrylate (TEGDMA) (<1 wt%), bisphenol A polyethylene glycol diether dimethacrylate (Bis-EMA)-6 (1–10 wt%), in addition to addition–fragmentation monomer (AFM), aromatic urethane dimethacrylate (AUDMA), and 1,12-dodecane dimethacrylate (DDDMA).
Filtek Supreme XTE Universal Shade: A2 body	N862133	Fillers: non-agglomerated nanosilica of 20 nm filler size and agglomerated zirconia/silica nanocluster with the size of 5–20 nm. The filler loading was 78.5 wt% (63.3% by volume). Organic matrix: Bis-GMA (5–10 wt%), UDMA (5–10 wt%), TEGDMA (5–10 wt%), Bis-EMA6 (1–10%), and polyethylene glycol dimethacrylate (PEGDMA) resins.

^1^ According to data provided by the manufacturer (3M OralCare, St. Paul, MN, USA).

**Table 2:** Mean ± standard deviation (SD) values of the bottom/top ratios’ Vickers hardness numbers (depth of cure) for different curing times and packing techniques.

Packing Techniques	Curing Time (Means ±SD)			*p*-Value
	20 s	30 s	40 s	
Filtek Supreme-Incremental (FS-I)	0.99 ± 0.17 ^Aa^	0.96 ± 0.01 ^Ba^	0.94 ± 0.02 ^Ba^	0.002 *
Filtek Bulk-Incremental (FB-I)	0.97 ± 0.04 ^Aa^	0.96 ± 0.02 ^Aa^	0.98 ± 0.03 ^Aa^	0.676 ^ns^
Filtek Bulk-Bulk (FB-B)	0.90 ± 0.02 ^Bb^	0.99 ± 0.04 ^Aa^	0.98 ± 0.03 ^ABa^	0.003 *
*p*-value	<0.001 *	0.054 ^ns^	0.111 ^ns^	

Different upper and lowercase superscript letters indicate statistically significant difference within the same row or column, respectively *; significant (*p* ≤ 0.05) ^ns^; non-significant (*p* > 0.05).

**Table 3:** Mean ± SD values of polymerization shrinkage for different packing techniques and curing times.

Packing Technique	Curing Time	Mean	St. Deviation
Filtek Supreme-Incremental (FS-I)	20 s	−1.9%	0.2
	30 s	−2.0%	0.3
	40 s	−1.9%	0.3
Filtek Bulk-Incremental (FB-I)	20 s	−1.6%	0.3
	30 s	−1.7%	0.4
	40 s	−1.7%	0.4
Filtek Bulk-Bulk (FB-B)	20 s	−1.4%	0.3
	30 s	−1.5%	0.3
	40 s	−1.5%	0.4

**Table 4:** Median (range) values of marginal leakage for different curing times and packing techniques.

Packing Technique	Curing Time			*p*-Value
	20 s	30 s	40 s	
Filtek Supreme-Incremental (FS-I)	2.0 (2.0)	1.0 (0.0)	1.6 (3.0)	0.72 ^ns^
Filtek Bulk Fill-Incremental (FB-I)	2.3 (2.0)	2.0 (2.0)	2.7 (3.0)	0.84 ^ns^
Filtek Bulk Fill-Bulk (FB-B)	1.4 (3.0)	2.0 (2.0)	1.4 (3.0)	0.82 ^ns^
*p*-value	0.21 ^ns^	0.05 *	0.19 ^ns^	

*; significant (*p* ≤ 0.05) ^ns^; non-significant (*p* > 0.05).

## Abbreviations

Bis-GMA: Bisphenol-A glycidyl dimethacrylate, UDMA: Urethane dimethacrylate, TEGDMA: Triethylene glycol dimethacrylate, Bis-EMA6: Bisphenol A polyethylene glycol diether dimethacrylate, AFM: Addition–fragmentation monomer, AUDMA: Aromatic urethane dimethacrylate, DDDMA: 1,12-dodecane dimethacrylate, PEGDMA: Polyethylene glycol dimethacrylate, wt%: % by weight.
